# Editorial: Retinal metabolism in health and disease

**DOI:** 10.3389/fopht.2024.1459318

**Published:** 2024-07-17

**Authors:** Sai Kocherlakota, James B. Hurley, Daisy Y. Shu

**Affiliations:** ^1^ Division of Eye and Vision, Department of Clinical Neuroscience, St. Erik Eye Hospital, Karolinska Institutet, Stockholm, Sweden; ^2^ Department of Biochemistry, University of Washington, Seattle, WA, United States; ^3^ School of Optometry and Vision Science, University of New South Wales, Sydney, NSW, Australia

**Keywords:** retina, metabolism, mitochondria, lactate, redox, prenylation, lipid, iron

## 1 Introduction

The human retina is a delicate, light-sensitive tissue lining the back of the eye, comprising an intricate network of cells organized in distinct layers. It can be divided into two primary components: the neural retina and the retinal pigment epithelium (RPE). The neural retina consists of various neuronal cells, including photoreceptors, interneurons, glial cells, astrocytes, and ganglion cells, all of which are essential for processing visual information. The RPE is integral to forming the blood-retinal barrier and plays many roles including absorbing scattered light, phagocytosing photoreceptor outer segments, transporting nutrients and waste, and supporting the metabolic and ionic demands of the retina.

The retina is one of the most metabolically active tissues in the body, and its functions are supported by nutrients delivered by the retinal pigment epithelium (RPE) through the highly fenestrated choroidal vasculature. Although the retina is an extension of the brain, its metabolic needs and the exchanges between its various cell types differ significantly from those of the brain. Photoreceptors predominantly convert glucose into lactate through aerobic glycolysis (Warburg effect), despite having abundant mitochondria. This high glucose utilization is facilitated by the RPE, which can use diverse fuels including amino acids, lactic acid and lipids for its energy production, thereby conserving glucose for the neural retina. This delicate metabolic balance is essential not only for proper vision but also for the survival and function of these post-mitotic cells.

The unique metabolic relationships between the RPE, photoreceptors, and other retinal cells are a fascinating area of investigation. This Research Topic brings together a collection of two original research articles providing new insights into the metabolic mechanisms underpinning retinal health and disease, as well as four review articles summarizing current progress in specific areas of retinal metabolism. Topics covered range from the role of lipid involvement in age-related macular degeneration (AMD) to metabolic roles of protein prenylation, lactate, iron chelation, cytosolic aspartate aminotransferase (GOT1), and the role of transmembrane proteins in mitochondrial dysfunction in retinal diseases. Through these contributions, we aim to advance our understanding of retinal metabolism and open new therapeutic avenues to treat blinding retinal diseases such as AMD and inherited retinal dystrophies.

Lipid dysregulation in the outer retina is particularly detrimental to overall retinal homeostasis with implications in many common retinal disorders including AMD. Subretinal drusenoid deposits (SDDs) have long been linked to high-risk phenotypes in AMD, but their exact compositions have remained elusive. Using advanced imaging mass spectrometry and nano liquid chromatography-tandem mass spectrometry (nLC-MS/MS), Anderson et al. mapped and identified lipids in SDDs, revealing how they are different from the adjacent photoreceptor outer segments. The findings offer insights into SDD formation and potential therapeutic targets for AMD.

Mitochondria and peroxisomes play a key role in cellular lipid metabolism. While the importance of mitochondria for retinal homeostasis has been studied extensively for a long time, the role of peroxisomes was only recently elucidated. In their review article, Landowski et al. discuss the roles of transmembrane protein 135 (TMEM135) in mitochondrial and peroxisomal functions and their implications for age-related retinal diseases, such as AMD. Identified through the study of mutant mice with accelerated retinal aging, TMEM135 regulates lipid metabolism and mitochondrial dynamics, and plays an important role in the export of docosahexaenoic acid (DHA) from peroxisomes. Dysfunction in TMEM135 leads to age-dependent retinal issues, underscoring its importance in retinal health. The review highlights the significance of TMEM135 in understanding molecular mechanisms of retinal aging and its potential as a therapeutic target for AMD.

Ashok et al. reviewed protein prenylation in the neural retina. Prenylation involves adding lipid groups to proteins, enabling their association with cell membranes. Recent findings reveal novel prenyltransferases, chaperones, and non-canonical prenylation motifs, and their implications for inherited retinal diseases, like Joubert syndrome and Leber congenital amaurosis. This review is timely and provides several elegant insights. Particularly notable is its delicate balance of highlighting the importance of computational approaches in predicting the cellular prenylome, while also reminding readers of the need for experimental validations of such predictions.

Subramanya et al. investigated the role of a cytosolic aspartate aminotransferase encoded by the glutamic-oxaloacetic transaminase 1 gene (*Got1*) in rod photoreceptors, focusing on its impact on retinal health. GOT1 enzyme is crucial for the malate-aspartate shuttle (MAS), essential for transferring reducing equivalents from the cytosol to the mitochondrial matrix, supporting photoreceptor metabolism and redox balance. Using a rod photoreceptor-specific *Got1* knockout mouse model, they observed that the loss of GOT1 led to a progressive photoreceptor degeneration, associated with increased retinal aspartate and NADH levels and altered expression of genes involved in various crucial metabolic pathways. The findings highlight the vital role that GOT1 plays in maintaining photoreceptor function and survival, suggesting that disruptions in GOT1 activity and MAS can significantly impair retinal health, leading to conditions such as retinitis pigmentosa.

Rajala et al. reviewed the multifaceted role of lactate in retinal cells. Lactate, traditionally seen as a metabolic byproduct, is now understood to have significant metabolic and non-metabolic functions. It serves as a key energy source for Müller cells, retinal ganglion cells (RGCs), and RPE cells, with the enzymes involved in lactate metabolism expressed throughout the retina. This review also highlights the signaling roles of lactate and provides interesting insights on the emerging field of histone lactylation, indicating its potential but underexplored role in retinal contexts. The review article elegantly argues for consideration of lactate as a key player in retinal health and its potential in therapeutic avenues.

Zhao et al. explore the impact of iron overload and chelation on bisretinoid levels in the retina. They induced iron accumulation through intravitreal injection of ferric ammonium citrate (FAC) in various mouse models. This elevated bisretinoid levels, which are associated with photoreceptor cell damage. Conversely, chelation therapy with deferiprone (DFP) reduces iron-catalyzed oxidation of bisretinoids, resulting in higher levels of A2E and A2-GPE. Their findings point toward the potential of iron chelation in mitigating retinal degeneration by reducing oxidative stress and preserving photoreceptor integrity.

Collectively, these contributions highlight the evolving understanding of retinal metabolism and its implications for retinal health and disease. The research presented not only advances our knowledge but also opens new avenues for therapeutic strategies aimed at metabolic reprogramming and the use of metabolites to counteract retinal degeneration. As the field progresses, it is imperative to continue exploring these complex metabolic networks and their interactions to develop effective interventions for retinal diseases. This Research Topic serves as a testament to the ongoing efforts and breakthroughs in retinal metabolism research, promising a future of improved diagnostic and therapeutic options for retinal disorders.

## Author contributions

SK: Writing – original draft, Writing – review & editing. JH: Writing – original draft, Writing – review & editing. DS: Writing – original draft, Writing – review & editing.

